# Synthesis of *cis*-hydrindan-2,4-diones bearing an all-carbon quaternary center by a Danheiser annulation

**DOI:** 10.3762/bjoc.14.237

**Published:** 2018-10-09

**Authors:** Gisela V Saborit, Carlos Cativiela, Ana I Jiménez, Josep Bonjoch, Ben Bradshaw

**Affiliations:** 1 Laboratori de Química Orgànica, Facultat de Farmàcia, IBUB, Universitat de Barcelona, Av. Joan XXIII s/n, 08028-Barcelona, Spain; 2 Departamento de Química Orgánica Instituto de Síntesis Química y Catálisis Homogénea (ISQCH), CSIC-Universidad de Zaragoza, 50009 Zaragoza, Spain

**Keywords:** alkaloid, Danheiser annulation, decahydroquinoline

## Abstract

A straightforward synthetic entry to functionalized hydrindane compounds based on a rapid assembly of the core nucleus by a Danheiser cycloaddition is reported. Valuable bicyclic building blocks containing the fused five and six-membered carbocyclic ring system can be achieved in only four steps from a simple acyclic β-keto ester.

## Introduction

**Figure 1:**
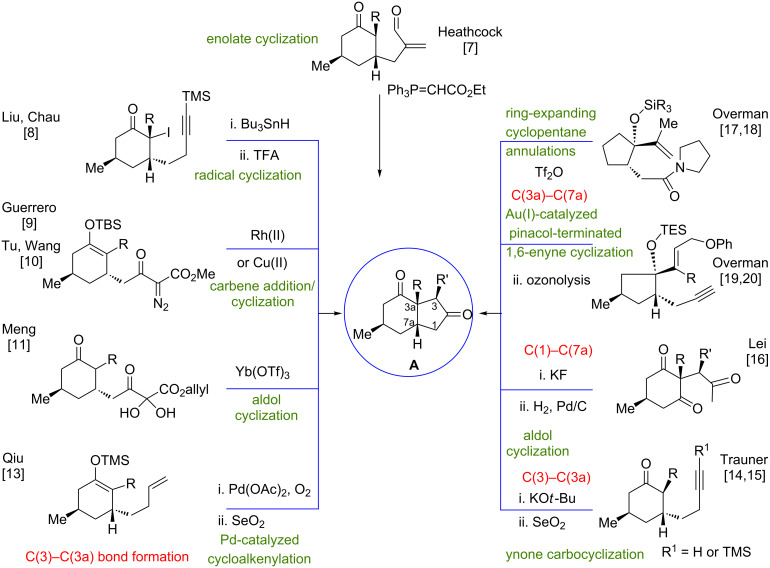
Previous synthetic approaches to 3a-substituted *cis*-hydrindan-2,4-diones.

*cis*-Fused hydrindanes (bicyclo[4.3.0]nonanes) [1–2], scaffolds of numerous natural products, are amenable to application as advanced intermediates in the total synthesis of *Lycopodium* alkaloids [3–6], 3a-substituted 2,4-dicarbonyl compounds being particularly useful in this field. The synthetic approaches toward these versatile building blocks (i.e., compounds with the functionalization pattern **A**) are outlined in Figure 1, which for the sake of clarity omits the substituents not involved in the bond-forming step in the final ring closure. Almost all the strategies developed to date involve the formation of the C3–C3a bond in the ring-closing step leading to the hydrindane nucleus. The carbocyclization takes place from polyfunctionalized cyclohexanones or related compounds through a Michael reaction [7], successive inter- and intramolecular radical processes [8], intramolecular carbene addition/cyclization [9–10], aldol cyclizations either under Lewis acid catalysis [11] or from diazoketones in the presence of bases (e.g., DBU) [12], Pd-catalyzed cycloalkenylation of a silyl enol ether [13], or base-promoted ynone carbocyclizations [14–15]. Another approach through an aldol cyclization, forming the C1–C7a bond instead, has also been reported [16]. Different strategies were developed by Overman through ring-expanding cyclopentane annulations based on a Prins–pinacol rearrangement [17–18] and Au(I)-catalyzed pinacol-terminated 1,6-enyne cyclizations [19–20], the C3a–C7a bond formation occurring in the last step of both procedures. Finally, Snyder gained access to the type **A** hydrindane nucleus from an acyclic compound by a cascade radical process using Mn(OAc)_3_ [21], although a *trans*-fused ring system was formed. Also of note, is the approach of Mori [22] to a dihydroindenedione based on an initial cyclization of an allyl iodide in a 1,3-cyclohexanedione side chain via an allyl anion (generated by Me_3_SiSnBu_3_ and CsF).

**Scheme 1:**
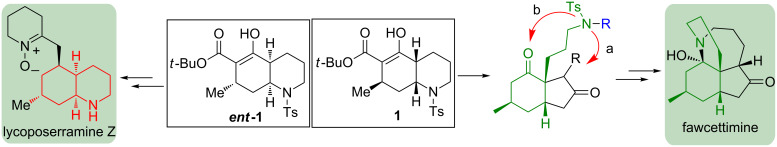
Decahydroquinoline **1** as a versatile building block for *Lycopodium* alkaloid synthesis.

**Figure 2:**
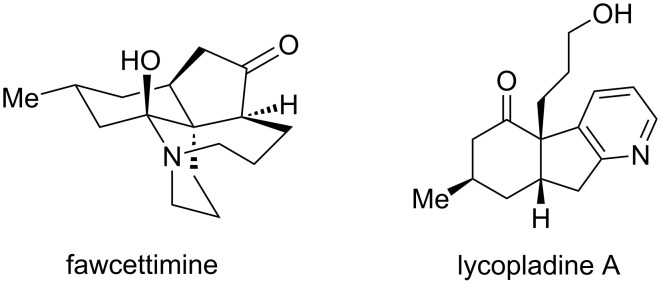
Examples of *Lycopodium* alkaloids synthesized from 3a-substituted hydrindan-2,4-diones.

As a continuation of our work on the synthesis of *Lycopodium* alkaloids [23–26], we hypothesized that decahydroquinoline **1**, a versatile building block for the synthesis of phlegmarine-type alkaloids, available in both enantiomeric forms, could also serve as an intermediate toward other *Lycopodium* alkaloids (e.g., fawcettimine). Thus, we surmised that building block **1** could be a new precursor of 3a-substituted hydrindan-2,4-diones (Scheme 1). This type of compounds, when adequately functionalized, has been used as advanced intermediates for the synthesis of the *Lycopodium* alkaloids carinatine A [11,14–15], 8-deoxyserratinine [10], fawcettidine [10], fawcettimine [7,10], lycojaponicumin C [10], lycopladine A [11,13–15], lycoposerramine R [13–14], and sieboldine [19–20] (Figure 2).

## Results and Discussion

Despite the potential of [3 + 2] cycloaddition reactions [27] to achieve *cis*-hydrindan-2,4-diones, their application to rapidly assemble the five-membered ring of the targeted 6,5-bicylic system has not been reported until now. Two examples using cycloaddition processes in this field should be mentioned: Diels–Alder [28] and Pauson–Khand [29] reactions have been used to build the hydrindane bicyclic system, but incorporating a different functionalization pattern in the cyclic compounds.

Proposed here is a new methodological approach to functionalized 3a-substituted hydrindane synthesis based on a Danheiser annulation involving a [3 + 2] cycloaddition reaction of a (trimethylsilyl)allene and a suitable cyclic α,β-unsaturated ketone [30–31]. Despite the applicability of the reaction to construct five-membered rings, it has not been extensively examined [32] for the synthesis of complex natural products.

**Scheme 2:**
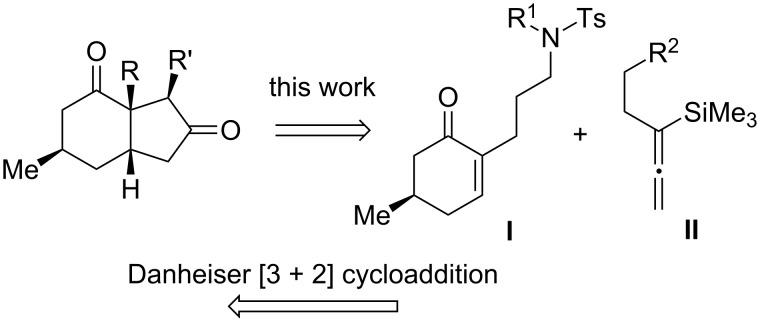
A de novo approach to 3a-substituted *cis*-hydrindan-2,4-diones.

As depicted in Scheme 2, the hydrindane core ring would be assembled by the simultaneous formation of two C–C bonds. This strategy is based on the disconnection across the C1–C7a and C3–C3a bonds, which according to the Danheiser annulation logic would reveal the cyclohexenone intermediate **I** and a silylallene such as **II**. The former, in turn, would disconnect back to β-keto ester **1** (Scheme 1), which we have employed in the synthesis of phlegmarine-type *Lycopodium* alkaloids [23–26].

**Scheme 3:**
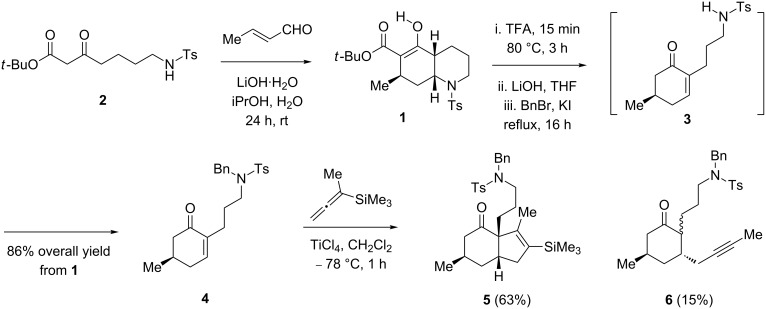
Synthesis of enone **4** and the Danheiser annulation. The depicted compounds are all racemic.

To prepare the starting material to evaluate the key Danheiser annulation reaction (Scheme 3), β-keto ester **2** was treated with crotonaldehyde and LiOH in iPrOH, following our previously developed procedure, to give decahydroquinoline **1** [23]. The removal of the *tert*-butyl ester group with TFA, followed by treatment with LiOH in refluxing THF promoted a retro-aza-Michael reaction yielding the ring-opened product **3** [33]. The latter was trapped in situ with benzyl bromide to furnish cyclohexenone **4** in 86% overall yield over the two steps from **1**. Additionally, the overall transformation from the starting material **2** was also performed in a one-pot sequence involving six reactions, namely, an intermolecular Michael reaction, aldol cyclization, intramolecular aza-Michael reaction, removal of a *tert*-butoxycarbonyl ester, base-promoted ring opening and tosylamide benzylation, without significant detrimental effect on the overall yield (see Supporting Information File 1).

**Scheme 4:**
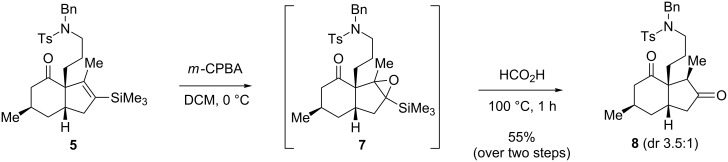
Transformation of the vinylsilane moiety to ketone **8**.

With the key precursor **4** in hand, the stage was set to study the Danheiser annulation step. Gratifyingly, treatment of **4** with TiCl_4_ in the presence of commercially available 1-methyl-1-(trimethylsilyl)allene at −78 °C for 1 h afforded the desired *cis*-6,5-bicylic core **5** in 63% yield as a single diastereomer. The desilylated product **6** was also obtained as a mixture of diastereomers. The high stereoselectivity of the ring-formation step can be explained by the suprafacial addition of the allene to the double bond of the α,β-unsaturated compound **4**, the diastereoselectivity being sterically controlled by the methyl group on the β-face. The transformation of the vinylsilane moiety in **5** into the corresponding carbonyl group (Scheme 4) was carried out by a two-step procedure involving epoxidation of the vinylsilane **5**, followed by a rearrangement of the diastereomeric mixture of epoxides **7** induced by formic acid [34–35]. The resulting ketone **8** was obtained as a 3.5:1 mixture of epimers at C3.

**Figure 3:**
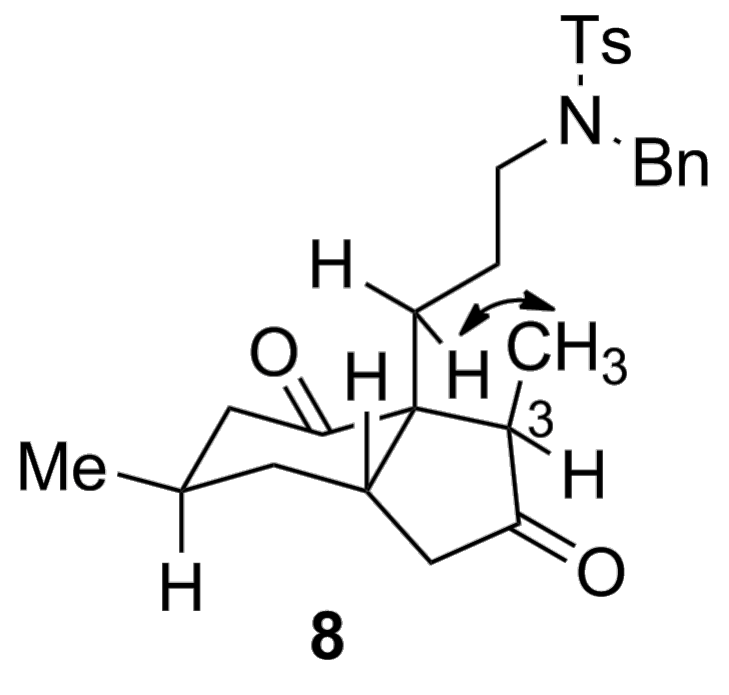
Stereoview of *cis*-hydrindane **8**.

The relative configuration of the major epimer of **8** (Figure 3) was established on the basis of a cross peak in the NOESY spectrum that correlated the methyl group at C3 with a methylene proton adjacent to the quaternary carbon of the side chain at C3a. The configuration at C3, adjacent to a carbonyl group, is not relevant for the potential application of this type of building block (i.e., **8**) in fawcettimine and related alkaloid synthesis, since this stereogenic center is epimerizable, as shown by Heathcock [7].

## Conclusion

In summary, we have successfully applied the Danheiser annulation reaction to rapidly assemble the 6,5-bicyclic nucleus of hydrindane compounds, expanding the usefulness of the versatile decahydroquinoline building block **1**. Although the compounds were prepared in racemic form, optically active substances are also accessible from enantiopure **1** [23]. The overall sequence shows the potential of this strategy to achieve functionalized hydrindanes, which may be useful for the synthesis of *Lycopodium* alkaloids embodying the aforementioned bicyclic core.

## Supporting Information

File 1 Experimental procedures and copies of ^1^H and ^13^C NMR spectra of all compounds.
