# Supplementary material for: Synthesis of cis-hydrindan-2,4-diones bearing an all-carbon quaternary center by a Danheiser annulation
Source: Beilstein J Org Chem. 2018 Oct 9;14:2597–601. doi: 10.3762/bjoc.14.237 (PMC6204772; doi:10.3762/bjoc.14.237)
Supplement: File 1 — Experimental procedures and copies of 1H and 13C NMR spectra of all compounds. [file Beilstein_J_Org_Chem-14-2597-s001.pdf]

Supporting Information

for

Synthesis of *cis*-hydrindan-2,4-diones bearing an *all*-carbon quaternary center by a Danheiser annulation

Gisela V. Saborit[1], Carlos Cativiela[2], Ana I. Jiménez[2], Josep Bonjoch*[1] and Ben Bradshaw*[1]

Address: [1]Laboratori de Química Orgànica, Facultat de Farmàcia, IBUB, Universitat de Barcelona, Av. Joan XXIII s/n, 08028-Barcelona, Spain and [2]Departamento de Química Orgánica Instituto de Síntesis Química y Catálisis Homogénea (ISQCH), CSIC-Universidad de Zaragoza, 50009 Zaragoza, Spain

Email address: Josep Bonjoch* - josep.bonjoch@ub.edu; Ben Bradshaw* - benbradshaw@ub.edu

*Corresponding author

Experimental procedures and copies of ^{1}H and ^{13}C NMR spectra of all compounds.

Table of contents

Experimental S2
^{1}H and ^{13}C NMR data of compounds **4–8** S6

General. All reactions were carried out under an argon atmosphere with dry, freshly distilled solvents under anhydrous conditions. All product mixtures were analyzed by thin-layer chromatography using TLC silica gel plates with a fluorescent indicator (λ = 254 nm). Analytical thin-layer chromatography was performed on SiO_2 (Merck silica gel 60 F_{254}), and the spots were located by UV light or/and a 1% $KMnO_4$ aqueous solution. Chromatography refers to flash chromatography and was carried out on SiO_2 (silica gel 60 ACC, 230–240 mesh). Drying of organic extracts during the reaction workup was performed over anhydrous Na_2SO_4. Chemical shifts of ^{1}H and ^{13}C NMR spectra are reported in ppm downfield (δ) from Me_4Si. All NMR data assignments are supported by gCOSY and gHSQC experiments.

5-Methyl-2-{3-[*N*-benzyl-*N*-(4-methylphenylsulfonyl)]aminopropyl} cyclohex-2-enone (4).

Method A: Decahydroquinoline **1** (5.0 g) was prepared from β-ketoester **2** in 79% yield on a 15 mmol scale, following our previously reported procedure.[1] Compound **1** (2.0 g, 4.75 mmol) was treated with TFA (3.3 mL) for 15 min at room temperature, the solvent was removed, and the last traces of TFA were removed by azeotroping with toluene (2 × 10 mL). The reaction flask was maintained on the rotatory evaporator in vacuo at 70 °C for 3 h. To a solution of the resulting ketone in THF (21 mL) were added successively LiOH (568 mg, 23.74 mmol, 5.0 equiv), KI (2.36 g, 14.24 mmol, 3.0 equiv) and benzyl bromide (1.7 mL, 14.24 mmol, 3.0 equiv), and the mixture was stirred at reflux temperature overnight. The mixture was allowed to reach room temperature, filtered through a pad of celite in vacuo and the filter cake was thoroughly washed with CH_2Cl_2. Volatiles were removed and the residue was purified by chromatography (2.5–10% EtOAc/hexane) to give cyclohexenone **4** (1.7 g, 86%, 68% overall yield from the starting material **2**); R_f = 0.42 (25% EtOAc/hexanes). ^{1}H NMR ($CDCl_3$, 400 MHz) δ 1.00 (d, J = 6.2 Hz, 3H, CH_3), 1.46 (quint, J = 7.7 Hz, 2H, H-2'), 1.90-2.06 (m, 4H, H-4, H-6, H-1'), 2.27-2.43

[1] Bradshaw, B.; Luque-Corredera, C.; Bonjoch, J. *Org. Lett.* **2013**, *15*, 326–329.

(m, 5H, H-4, H-6, ArCH_3), 3.03 (m, 2H, H-3'), 4.30 (CH_2Ph), 6.46 (m, 1H, H-3), 7.28 (m, 7H, *o*-Ts, Ph), 7.71 (d, *J* = 8.3 Hz, 2H, *m*-Ts); ^{13}C NMR ($CDCl_3$, 100 MHz) δ 21.2 (CH_3), 21.6 (ArCH_3), 26.5 (C-5), 26.7 (C-2'), 30.6 (C-1'), 34.4 (C-4), 46.6 (C-6), 47.6 (C-3'), 51.9 (CH_2Ph), 127.3 (*o*-Ts), 127.8 (*p*-Bn), 128.6 (*o*-Bn), 129.8 (*m*-Ts), 136.6 (*ipso*-Bn), 137.0 (*p*-Ts), 138.2 (*ipso*-Ts), 143.3 (C-2), 145.1 (C-3), 199.5 (C-1). HRMS (ESI-TOF) *m/z*: $[M + H]^+$ Calcd for $C_{24}H_{30}NO_3S$ 412.1941, found 412.1938.

Method B (one-pot procedure): To a cooled (0 °C) solution of keto ester **2** (1.05 g, 2.84 mmol, 1.0 equiv) in iPrOH (10 mL) and H_2O (0.5 mL, 28.4 mmol, 10 equiv) was added crotonaldehyde (0.26 mL, 3.13 mmol, 1.1 equiv) followed by LiOH·H_2O (119 mg, 2.84 mmol, 1 equiv). The reaction mixture was allowed to reach room temperature and stirred for 24 h. To this flask was added the resin (PS) of *p*-toluenesulfonic acid (4.3 g, 30–60 mesh, Aldrich) and the mixture was stirred for 2 h. After filtration and concentration the crude toluenesulfonamide **3** was processed as in Method A to give **4** (674 mg, 56% from **2**).

(1*RS*,4*SR*,6*RS*)-1-[*N*-Benzyl-*N*-(4-methylphenylsulfonyl)-3-aminopropyl]-4,9-dimethyl-8 trimethylsilylbicyclo[4.3.0]non-8-en-2-one (5). To a cooled (−78 °C) solution of **4** (471 mg, 1.14 mmol) and 1-methyl-1-(trimethylsilyl)allene (0.32 mL, 1.94 mmol) in CH_2Cl_2 (6 mL), $TiCl_4$ (0.21 mL, 1.7 mmol) was added dropwise. The resulting dark red solution was stirred at that temperature for 1 h and then transferred to a mixture of water (25 mL) and diethyl ether (25 mL). The aqueous layer was extracted with diethyl ether (3 × 25 mL) and the combined organic layers were washed with brine (25 mL), dried, and concentrated. The crude product was purified by chromatography (2.5–10% EtOAc/hexane) to give **5** (385 mg, 63%), followed by **6** (78 mg, 15%), as an epimeric mixture.

Compund **5**: R_f = 0.54 (25% EtOAc/hexanes); ^{1}H NMR ($CDCl_3$, 400 MHz) δ 0.09 (s, 9H, $(CH_3)_3$Si), 0.85 (d, *J* = 6.8 Hz, 3H, CH_3), 1.15-1.25 (m, 4H, H-5eq, H-1', 2H-2'), 1.39 (td, *J* = 12.4, 3.3 Hz, 1H, H-5ax), 1.44 (t, *J* = 2.1 Hz, 3H, 9-CH_3), 1.50 (dm, *J* = 13.6 Hz, 1H, H-1'), 1.77 (dd, *J* = 16.0, 8.5 Hz, 1H, H-3ax), 1.99-2.05 (m, 2H, H-4, H-7), 2.11 (dddd, *J* = 10, 5, 5, 5 Hz, 1H, H-6), 2.27 (ddd, *J* = 16.0, 5.6, 1.4 Hz, 1H, H-3eq), 2.43 (s, 3H, ArCH_3), 2.44 (ddq, *J* = 11.0, 5.5, 2.1

Hz, 1H, H-7), 3.06 (t, *J* = 7.4 Hz, 2H, H-3'), 4.30 (2d, *J* = 14.8 Hz, 2H, CH_2Ph), 7.30-7.35 (m, 7H, *o*-Ts, Ph), 7.73 (d, *J* = 8.3 Hz, 2H, *m*-Ts); ^{13}C NMR ($CDCl_3$, 100 MHz) δ -0.6 ($(CH_3)_3Si$), 14.0 (9-CH_3), 21.6 (CH_3), 21.6 ($ArCH_3$), 23.4 (C-2'), 26.9 (C-4), 31.0 (C-5), 37.0 (C-1'), 41.6 (C-6), 43.5 (C-7), 47.3 (C-3), 49.0 (C-3'), 52.4 (NCH_2Ar), 69.7 (C-1), 127.3 (*o*-Ts), 127.8 (Ph), 128.4 (Ph), 128.7 (Ph), 129.8 (*m*-Ts), 136.8 (*ipso*-Ph), 137.1 (*p*-Ts), 138.7 (*ipso*-Ts), 143.3 (C-8), 148.8 (C-9), 214.7 (C-2). HRMS (ESI-TOF) *m/z*: $[M + H]^+$ Calcd for $C_{31}H_{44}NO_3SSi$ 538.2806, found 538.2798.

Data for 3-(2-butynyl)-5-methyl-2-[(*N*-benzyl-*N*-(4-methylphenylsulfonyl)-3-aminopropyl]cyclohexanone (**6**): R_f = 0.39 (25% EtOAc/hexanes); ^1H NMR ($CDCl_3$, 400 MHz, major isomer) δ 0.99 (d, *J* = 6.8 Hz, 5-CH_3), 1.14-1.54 (m, 4H, H-1' 2H-2', H-4), 1.63 (2dd, *J* = 11.0, 2.6 Hz, 1H, H-1'), 1.75 (t, *J* = 2.4 Hz, CH_3), 1.93 (m, 1H, H- 1''), 2.07 (m, 3H, H-1', H-4, H-5), 2.26 (m, 2H, H-3, H-6, H-2), 2.43 (s, $ArCH_3$), 3.06 (m, 2H, H-3'), 4.23 (s, 2H, CH_2Ph), 7.29 (m, 5H, Ph, *m*-Ts), 7.71 (d, *J* = 8.0 Hz, 2H *o*-Ts). ^{13}C NMR ($CDCl_3$, 100 MHz) δ 3.6 (CH_3), 18.0 (C-1´´), 21.6 (CH_3Ts), 22.4 (5-CH_3), 23.2 (C-1´), 26.1 (C-2´), 29.8 (C-5), 38.7 (C-4), 40.2 (C-3), 47.6 (C-3´), 50.2 (C-6), 51.4 (NCH_2Ar), 52.6 (C-2), 76.5 (C-3´´), 77.8 (C-2´´), 127.3 (*o*-Ts), 127.8 (*p*-Bn), 128.5 (*m*-Bn), 128.6 (*o*-Bn), 129.8 (*m*-Ts), 136.6 (*ipso*-Bn), 136.7 (*p*-Ts), 137.1 (*ipso*-Ts), 143.3 (*p*-Ts), 211.8 (C-1). HRMS (ESI-TOF) *m/z*: $[M + H]^+$ Calcd for $C_{28}H_{36}NO_3S$ 466.2410, found 466.2415.

(1*RS*,4S*R*,6*RS*,9*SR*)-1-[*N*-Benzyl-*N*-(4-methylphenylsulfonyl)-3-aminopropyl]-4,9-dimethylbicyclo[4.3.0]non-2,8-dione (8). To a solution of **5** (30 mg, 0.056 mmol) in CH_2Cl_2 (1 mL) was added $NaHCO_3$ (9 mg, 0.112 mmol) followed by *m*-CPBA (12 mg, 0.053 mmol) at 0 °C. After stirring for 1 h at 0 °C, 2-methyl-2-butene (0.12 mL) was added to quench the remaining traces of *m*-CPBA and the mixture was allowed to stir for 10 min at 0 °C. Filtration and concentration afforded a 3:1 mixture of diastereomeric epoxy silanes **7**, which was directly used in the following step without any further purification. An analytical sample of **7** was obtained by chromatography (5–25% EtOAc in hexanes): ^1H NMR ($CDCl_3$, 400 MHz) δ 0.08 (s, 9H, $(CH_3)_3Si$), 0.95 (d, *J* = 6.4 Hz, 3H, CH_3), 1.24 (m, 2H, H-1', H-5), 1.35 (s, 3H, 9-CH_3), 1.38-1.67 (m, 6H,

2H-2’, 2H-7, H-5, H-1’), 1.84 (m, 2H, H-4, H-6), 1.95 (m, 2H, H-3), 3.06 (m, 2H, H-3’), 4.25 and 4.33 (2d, *J* = 14.8 Hz, 1H each, CH_2Ph), 7.32 (m, 7H, *o*-Ts, Ph), 7.73 (d, 2H, *J* = 8.0 Hz, *m*-Ts); ^{13}C NMR ($CDCl_3$, 100 MHz) δ −2.3 ($(CH_3)_3Si$), 16.2 (9-CH_3), 21.6 ($ArCH_3$), 22.4 (CH_3), 23.0 (C-2’), 28.0 (C-7), 31.4 (C-6), 31.6 (C-5), 33.3 (C-1’), 37.1 (C-4), 47.9 (C-3), 48.9 (C-3’), 52.4 (CH_2Ph), 59.5 (C-1), 61.4 (C-9), 69.3 (C-8), 127.3 (*o*-Ts), 127.9 (*p*-Bn), 128.7 (*m*-Bn), 128.8 (*o*-Bn), 129.9 (*m*-Ts), 136.5 (*ipso*-Bn), 137.0 (*p*-Ts), 143.4 (*ipso*-Ts), 214.7 (C-2), 217.8 (C-9). HRMS (ESI-TOF) *m/z*: $[M + H]^+$ Calcd for $C_{31}H_{44}NO_4SSi$ 554.2755, found 554.2744.

A solution of the crude epoxide **7** in formic acid (1.0 mL) was heated at reflux for 1 h, cooled, concentrated, and purified by chromatography (2.5–10% EtOAc in hexanes) to afford diketone **8** as a 3.5:1 mixture of diastereomers (15 mg, 55% yield, over 2 steps). Data for the major diastereomer: R_f = 0.23 (50% EtOAc/hexanes); ^1H NMR ($CDCl_3$, 400 MHz) δ 0.75 (d, *J* = 7.6 Hz, 3H, 9-CH_3), 0.97 (d, *J* = 6.4 Hz, 3H, 4-CH_3), 0.99 (m, 1H, H-2’), 1.04 (dd, *J* = 6.8, 4.0 Hz, 1H, H-2'), 1.36 (m, 2H, H-1'), 1.63-1.71 (m, 2H, H-5), 1.91-2.01 (m, 2H, H-7eq, H-3ax), 2.11 (m, 1H, H-4), 2.17 (m, 1H, H-7ax), 2.29 (dd, *J* = 13.6, 4.0 Hz, 2H, H-3eq), 2.45 (masked, 1H, H-6), 2.77 (q, *J* = 7.6 Hz, 1H, H-9), 2.97 (m, 1H, H-3’), 3.09 (q, *J* = 7.2 Hz, 1H, H-3'), 4.12-4.33 (2d, *J* = 14.8 Hz, 2H, CH_2Ph), 7.30-7.35 (m, 6H, *m*-Ts, Ph), 7.73 (d, *J* = 8.3 Hz, 2H, *o*-Ts); ^{13}C NMR ($CDCl_3$, 100 MHz) δ 9.9 (9-CH_3), 21.1 (4-CH_3), 21.7 (CH_3Ar), 23.8 (C-2'), 28.0 (C-1'), 29.9 (C-4), 32.5 (C-5), 38.6 (C-6), 40.7 (C-7), 45.5 (C-3), 46.5 (C-9), 49.0 (C-3'), 53.0 (NCH_2Ar), 58.1 (C-1), 127.3 (*o*-Ts), 128.2 (Ph), 128.7 (Ph), 128.8 (Ph), 129.9 (*m*-Ts), 136.5 (*ipso*-Ph), 136.6 (*p*-Ts), 143.6 (*ipso*-Ts), 212.6 (C-8), 217.8 (C-2). HRMS (ESI-TOF) *m/z*: $[M + H]^+$ Calcd for $C_{28}H_{36}NO_4S$ 482.2360, found 482.2359.

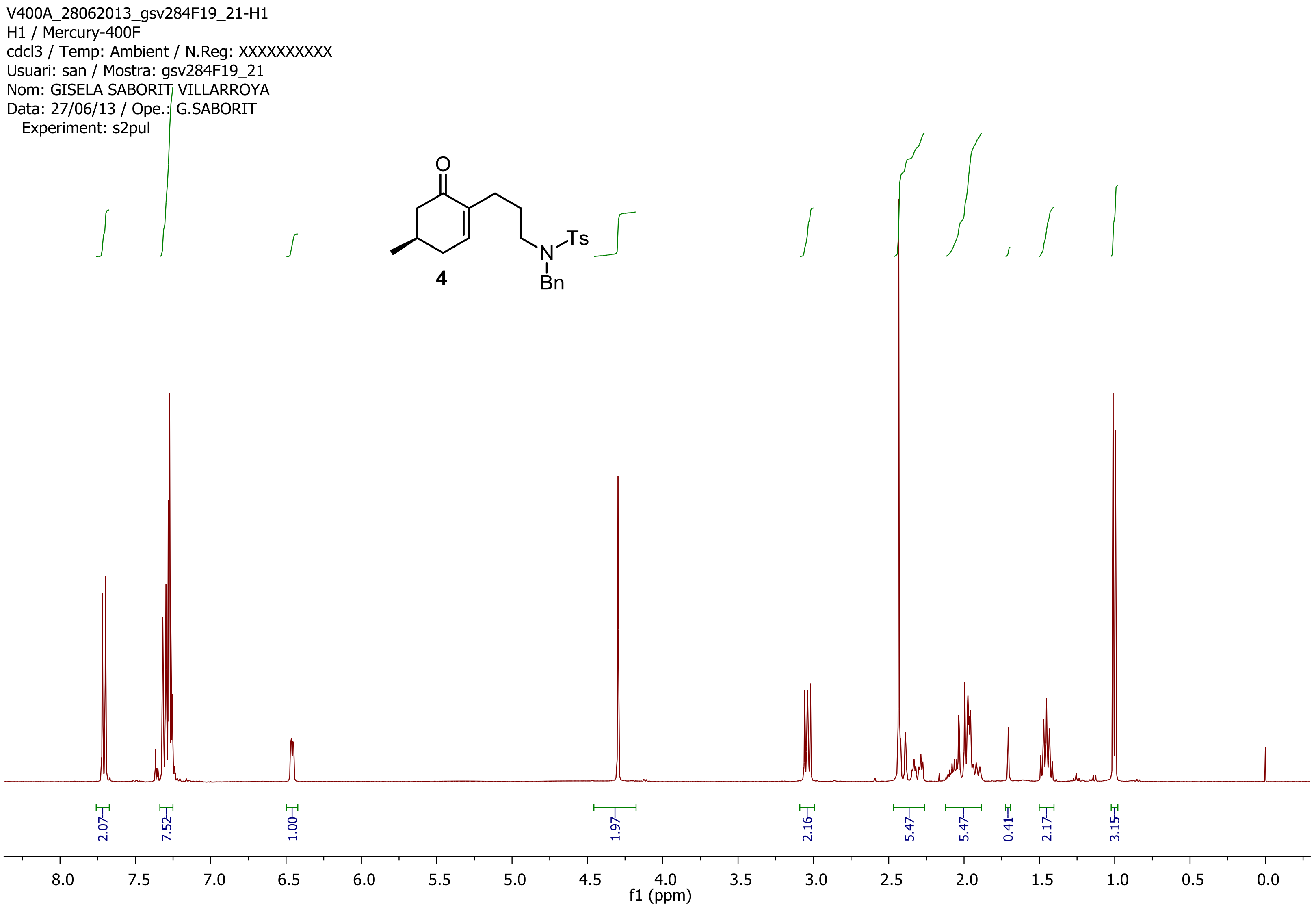
V400A_28062013_gsv284F19_21-H1
H1 / Mercury-400F
cdcl3 / Temp: Ambient / N.Reg: XXXXXXXXXX
Usuari: san / Mostra: gsv284F19_21
Nom: GISELA SABORIT VILLARROYA
Data: 27/06/13 / Ope.: G.SABORIT
Experiment: s2pul
O
Ts
N
Bn
4
2.07
7.52
1.00
1.97
2.16
5.47
5.47
0.41
2.17
3.15
8.0
7.5
7.0
6.5
6.0
5.5
5.0
4.5
4.0
3.5
3.0
2.5
2.0
1.5
1.0
0.5
0.0
f1 (ppm)

V400A_28062013_gsv284F19_21-C13
H1 / Mercury-400F
cdcl3 / Temp: Ambient / N.Reg: XXXXXXXXXX
Usuari: san / Mostra: gsv284F19_21
Nom: GISELA SABORIT VILLARROYA
Data: 27/06/13 / Ope.: G.SABORIT
Experiment: s2pul

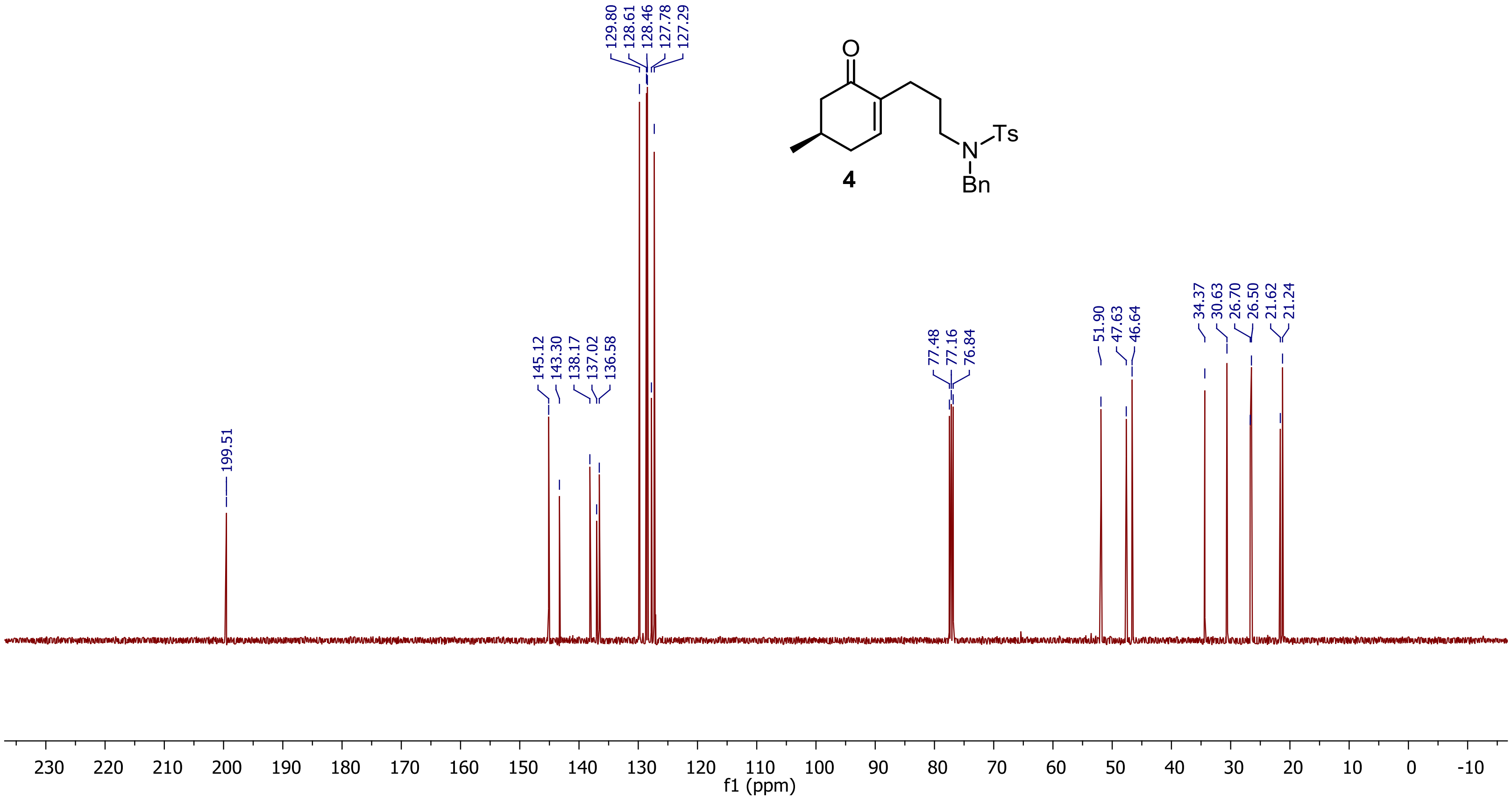

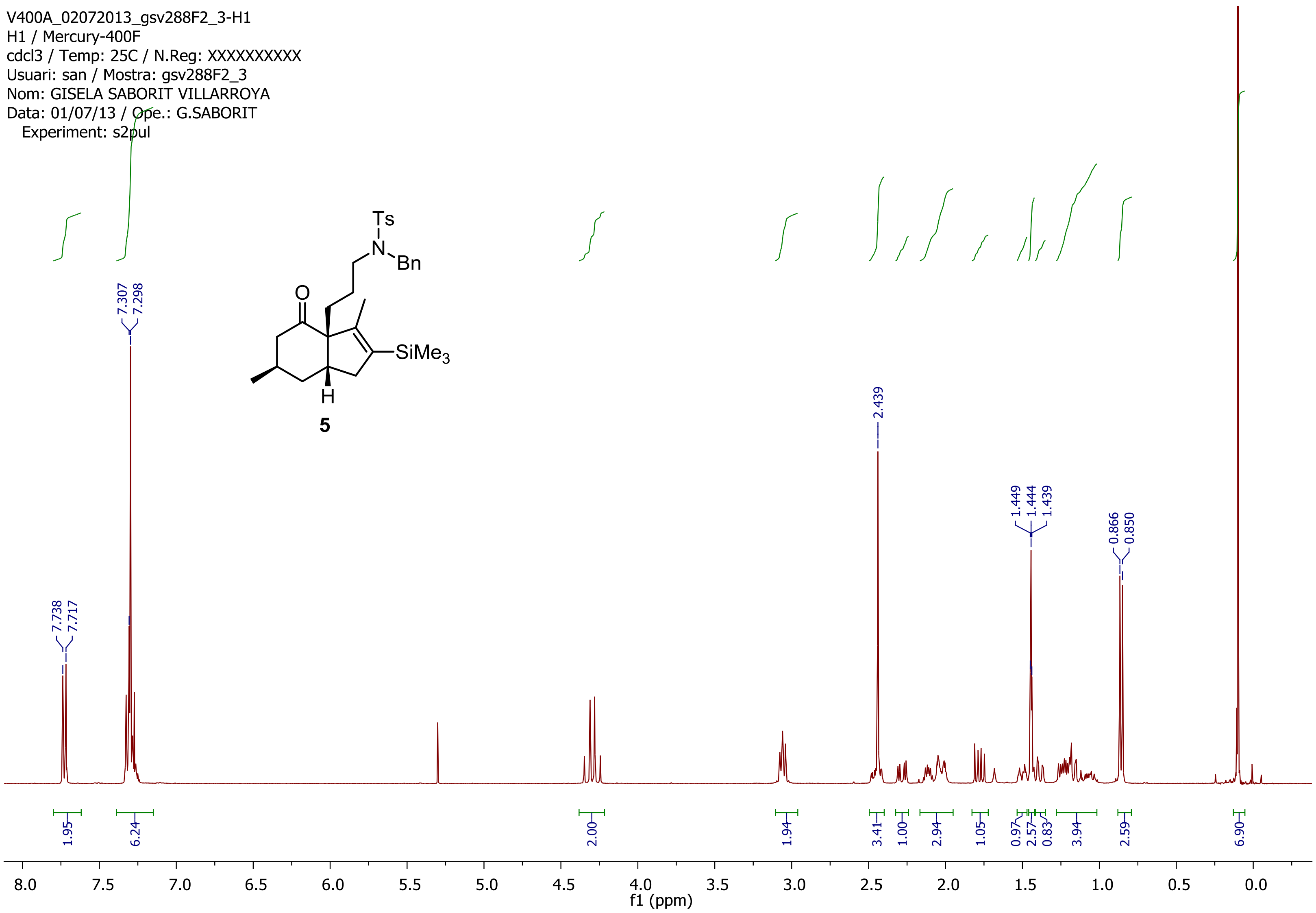
V400A_02072013_gsv288F2_3-H1
H1 / Mercury-400F
cdcl3 / Temp: 25C / N.Reg: XXXXXXXXXX
Usuari: san / Mostra: gsv288F2_3
Nom: GISELA SABORIT VILLARROYA
Data: 01/07/13 / Ope.: G.SABORIT
Experiment: s2pul
Ts
N
Bn
O
SiMe3
H
5
7.738
7.717
7.307
7.298
2.439
1.449
1.444
1.439
0.866
0.850
1.95
6.24
2.00
1.94
3.41
1.00
2.94
1.05
0.97
2.57
0.83
3.94
2.59
6.90
8.0 7.5 7.0 6.5 6.0 5.5 5.0 4.5 4.0 3.5 3.0 2.5 2.0 1.5 1.0 0.5 0.0
f1 (ppm)

V400A_02072013_gsv288F2_3-C13
H1 / Mercury-400F
cdcl3 / Temp: 25C / N.Reg: XXXXXXXXXX
Usuari: san / Mostra: gsv288F2_3
Nom: GISELA SABORIT VILLARROYA
Data: 01/07/13 / Ope.: G.SABORIT
Experiment: s2pul

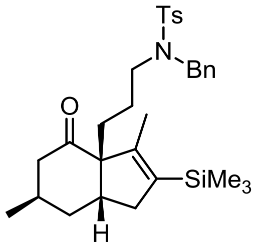

5

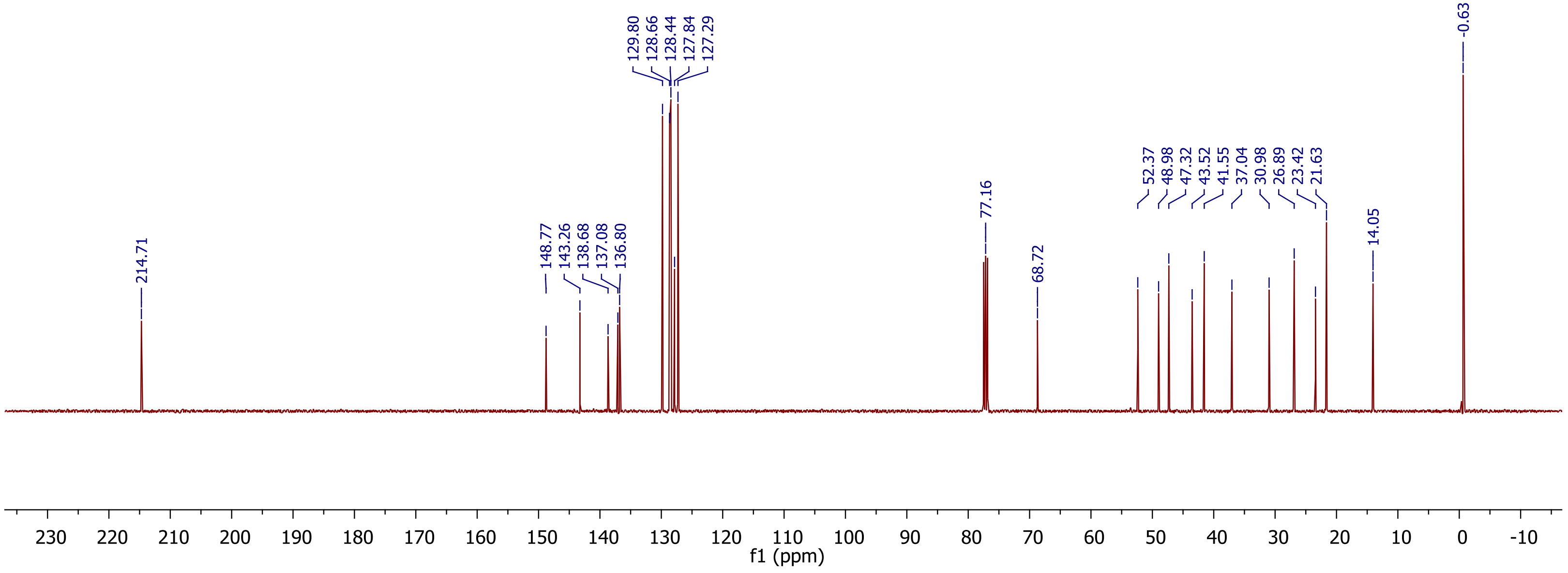

V400A_03072013_gsv288F5_9-H1
H1 / Mercury-400F
cdcl3 / Temp: 25C / N.Reg: XXXXXXXXXX
Usuari: san / Mostra: gsv288F5_9
Nom: GISELA SABORIT VILLARROYA
Data: 03/07/13 / Ope.: G.SABORIT
Experiment: s2pul

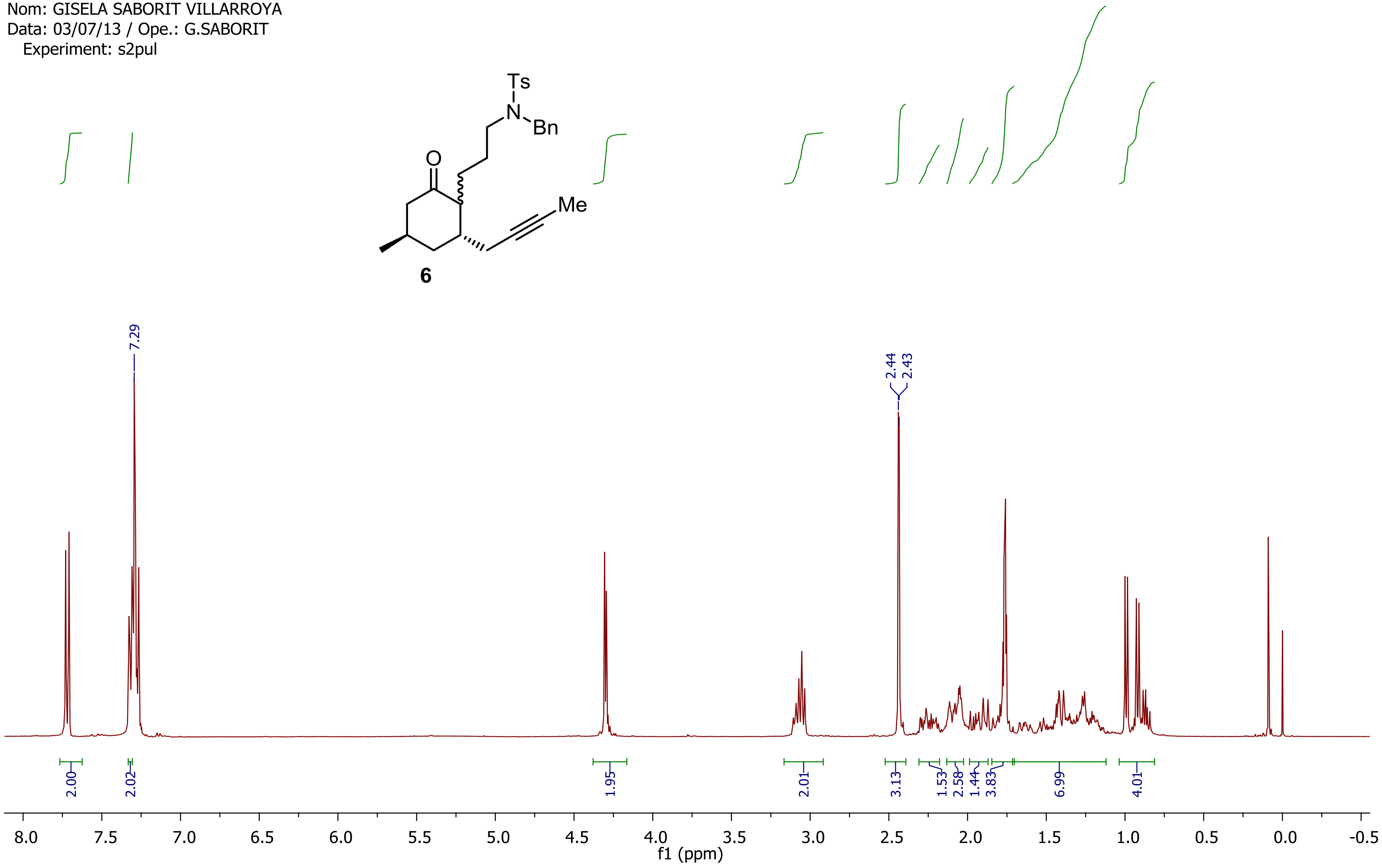

V400A_03072013_gsv288F5_9-C13
H1 / Mercury-400F
cdcl3 / Temp: 25C / N.Reg: XXXXXXXXXX
Usuari: san / Mostra: gsv288F5_9
Nom: GISELA SABORIT VILLARROYA
Data: 03/07/13 / Ope.: G.SABORIT
Experiment: s2pul

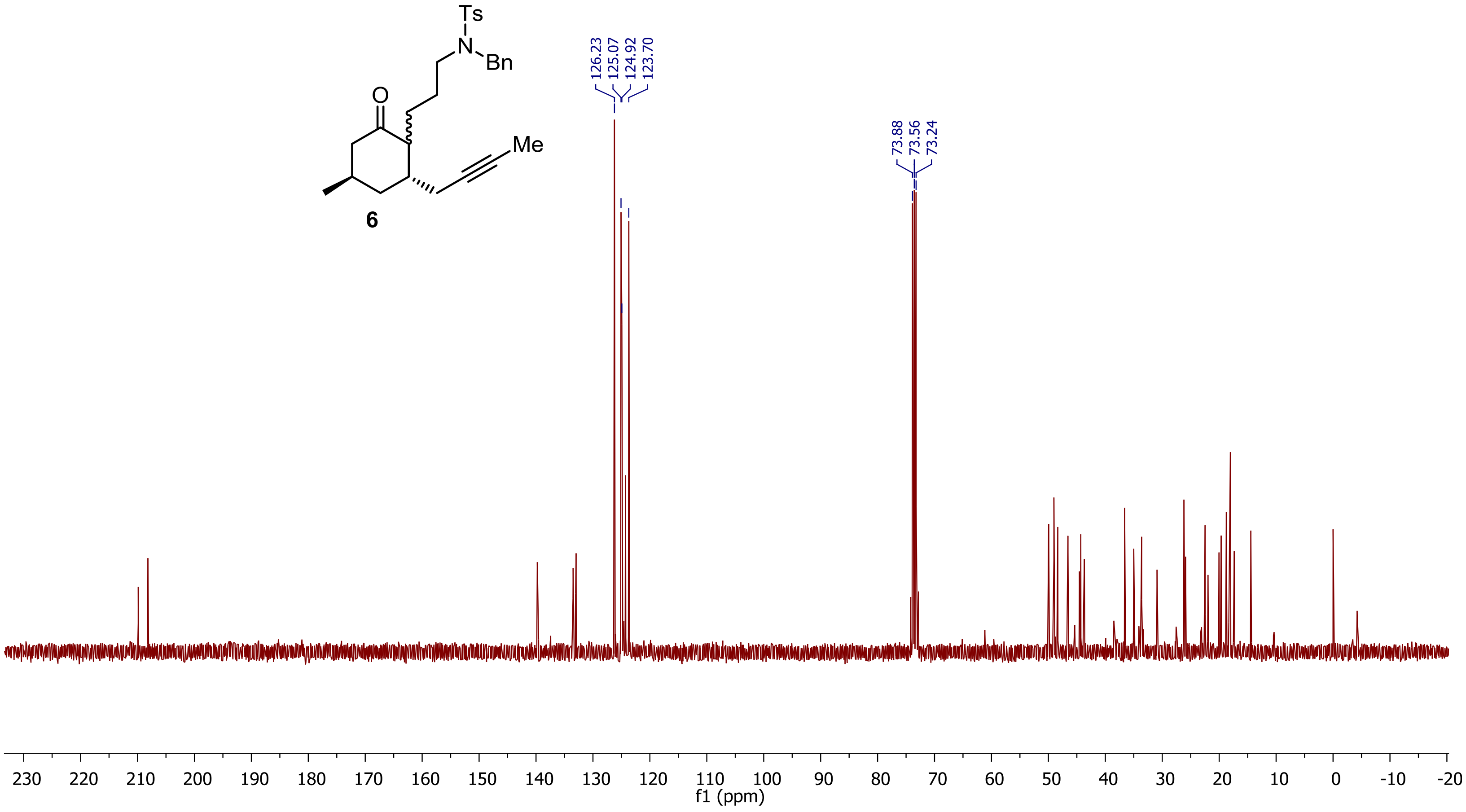

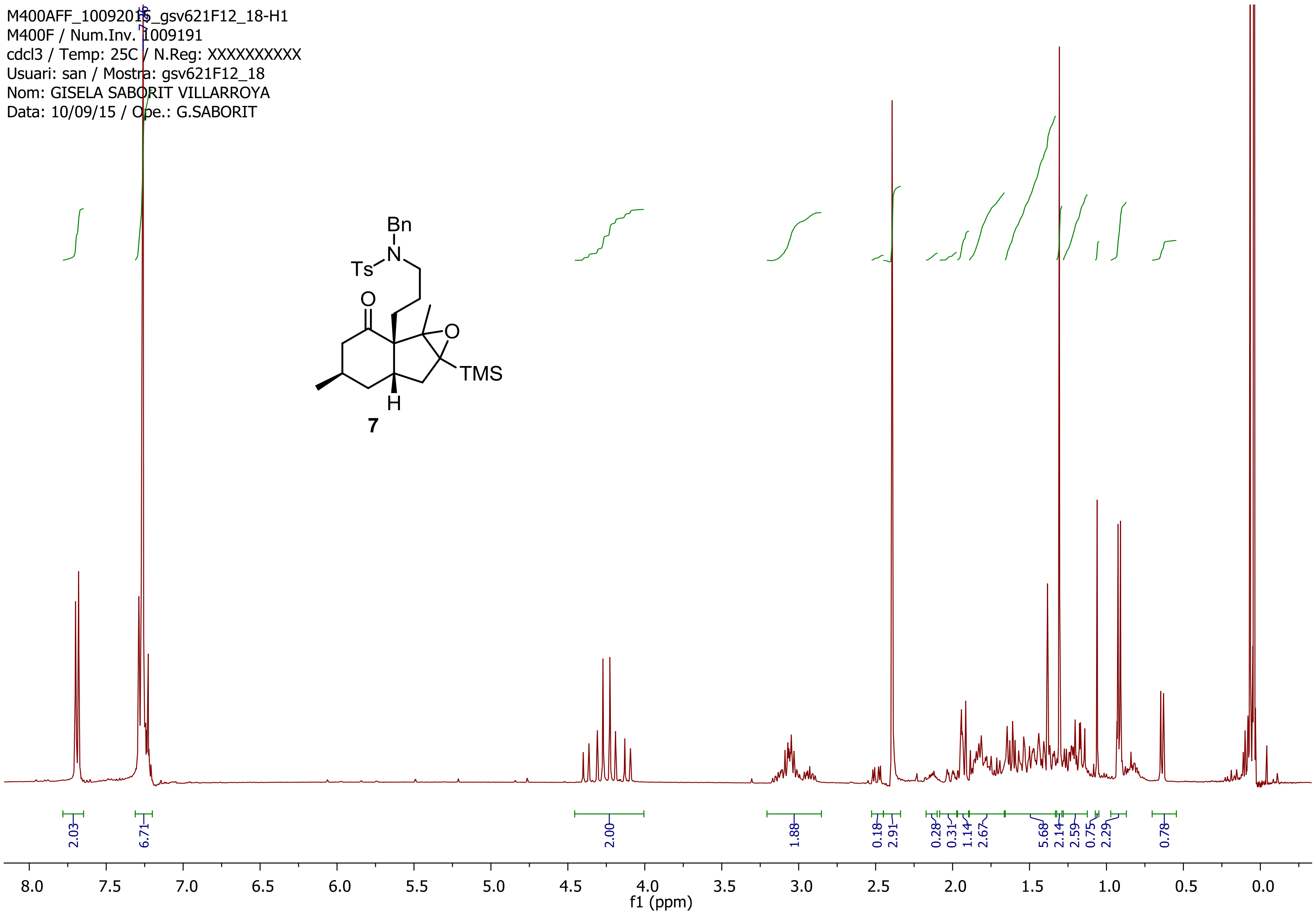
M400AFF_10092015_gsv621F12_18-H1
M400F / Num.Inv. 1009191
cdcl3 / Temp: 25C / N.Reg: XXXXXXXXXX
Usuari: san / Mostra: gsv621F12_18
Nom: GISELA SABORIT VILLARROYA
Data: 10/09/15 / Ope.: G.SABORIT
Bn
N
Ts
O
O
TMS
H
7
2.03
6.71
2.00
1.88
0.18
2.91
0.28
0.31
1.14
2.67
5.68
2.14
2.59
0.75
2.29
0.78
f1 (ppm)

V400A_19052014_gsv413_carboni-C13
H1 / 400
cdcl3 / Temp: Ambient / N.Reg: XXXXXXXXXX
Usuari: san / Mostra: gsv413_carboni
Nom: GISELA SABORIT VILLARROYA
Data: 19/05/14 / Ope.: G.SABORIT
Experiment: s2pul

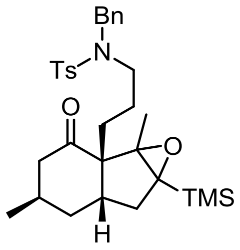

7

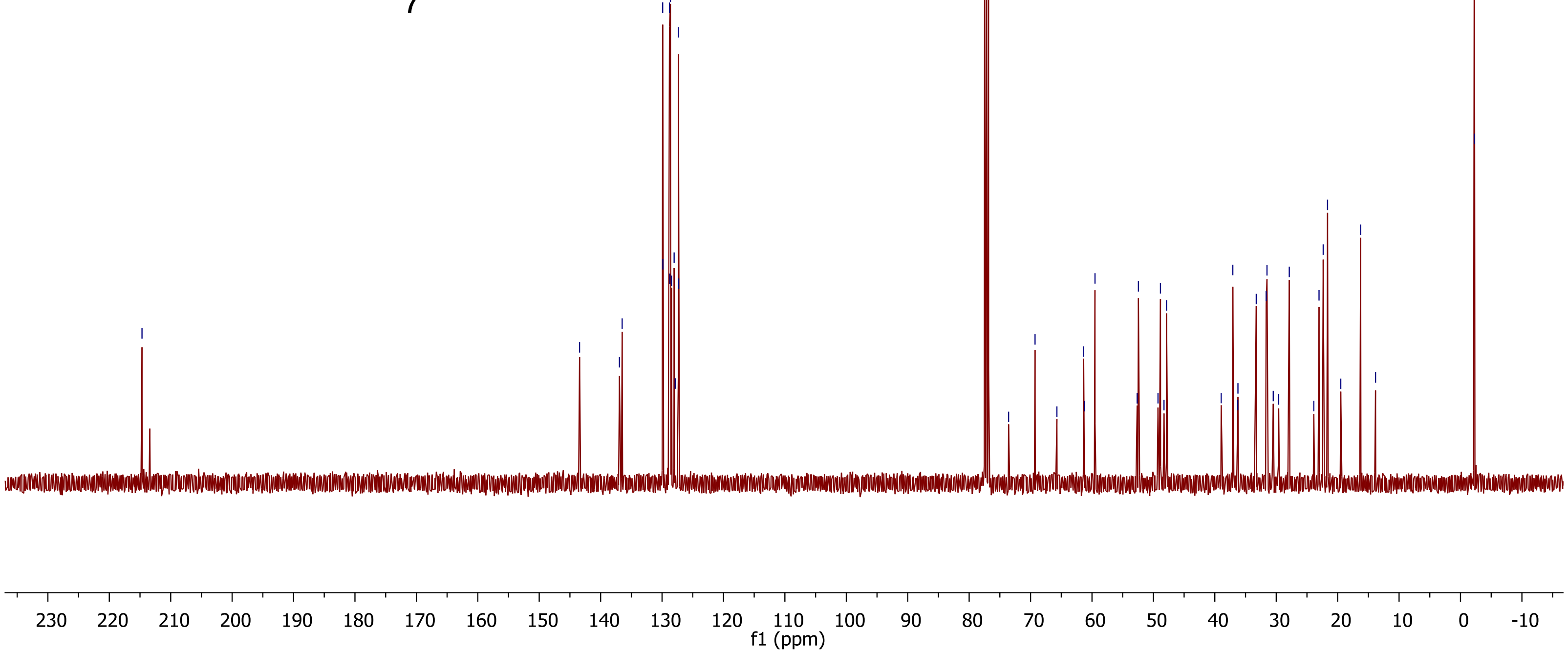

M400AFF_22052014_gsv413_base-H1
Submitq / 400
cdcl3 / Temp: 25C / N.Reg: XXXXXXXXXX
Usuari: san / Mostra: gsv413_base
Nom: GISELA SABORIT VILLARROYA
Data: 22/05/14 / Ope.: G.SABORIT

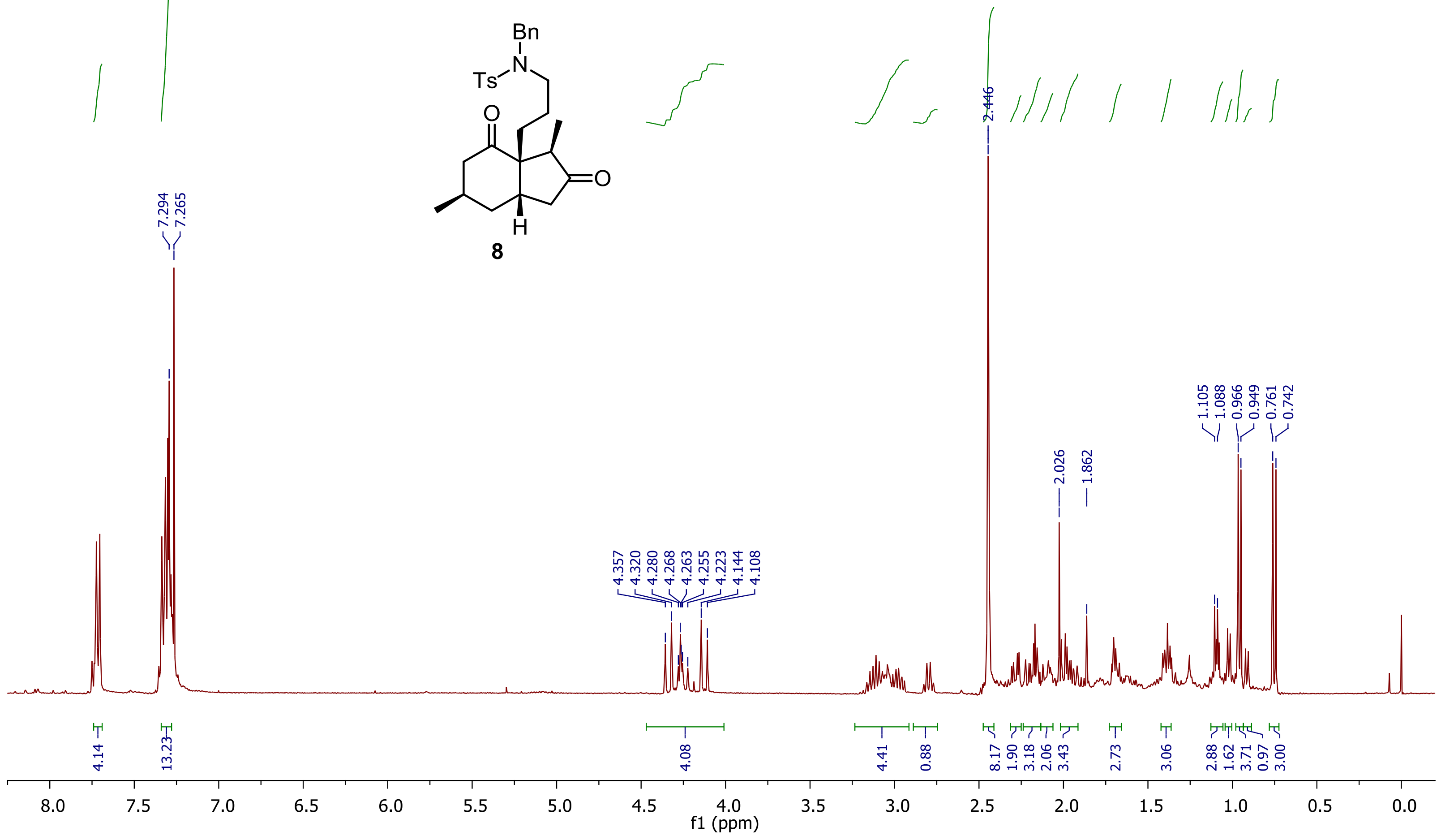

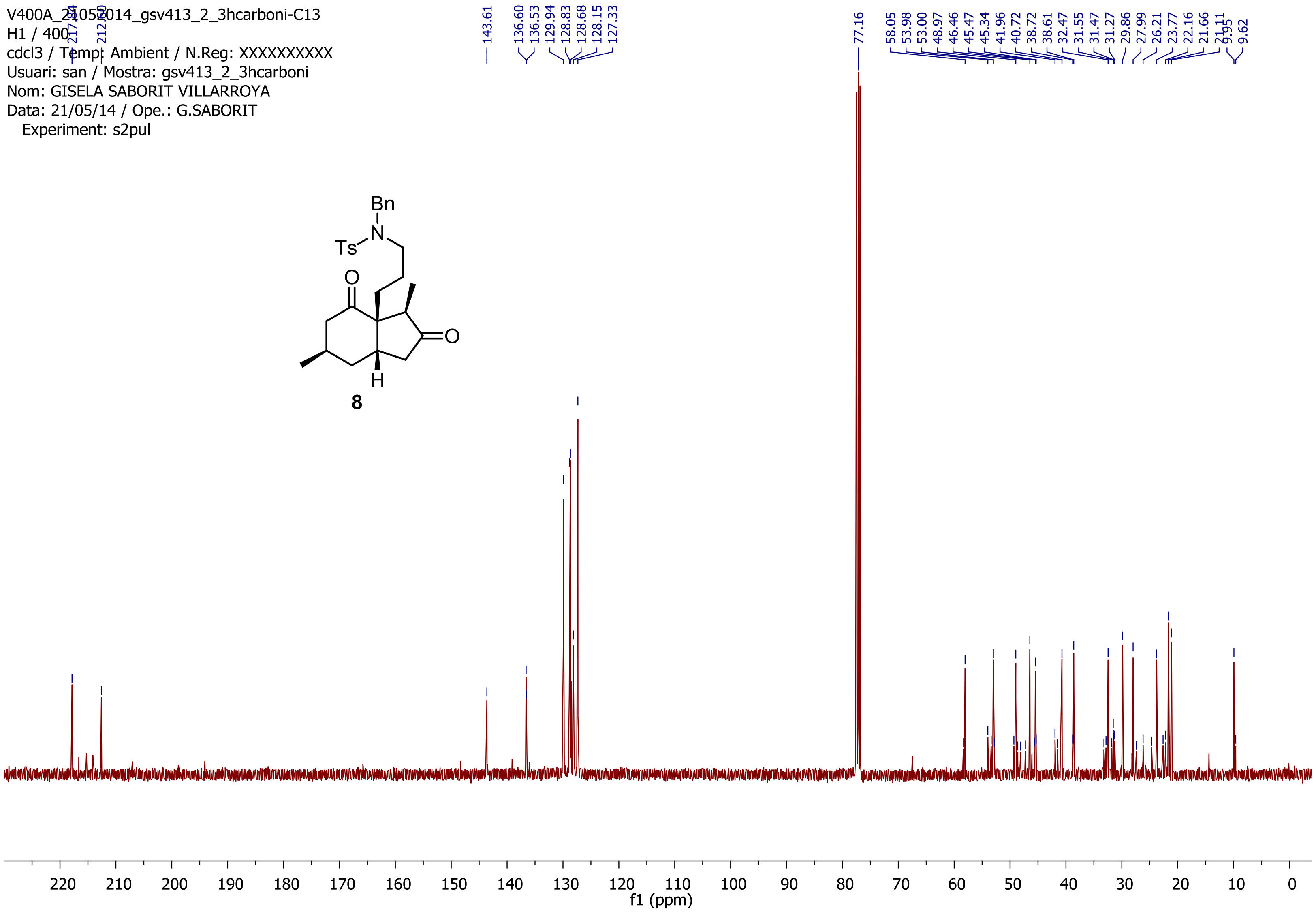

V400A_21052014_gsv413_2_3hcarboni-C13
H1 / 400
cdcl3 / Temp: Ambient / N.Reg: XXXXXXXXXX
Usuari: san / Mostra: gsv413_2_3hcarboni
Nom: GISELA SABORIT VILLARROYA
Data: 21/05/14 / Ope.: G.SABORIT
Experiment: s2pul
217.84
212.60
143.61
136.60
136.53
129.94
128.83
128.68
128.15
127.33
77.16
58.05
53.98
53.00
48.97
46.46
45.47
45.34
41.96
40.72
38.72
38.61
32.47
31.55
31.47
31.27
29.86
27.99
26.21
23.77
22.16
21.66
21.11
9.95
9.62
Bn
N
Ts
O
O
H
8
220
210
200
190
180
170
160
150
140
130
120
110
100
90
80
70
60
50
40
30
20
10
0
f1 (ppm)
